# Analgesia linked to Nav1.7 loss of function requires µ- and δ-opioid receptors

**DOI:** 10.12688/wellcomeopenres.14687.1

**Published:** 2018-08-16

**Authors:** Vanessa Pereira, Queensta Millet, Jose Aramburu, Cristina Lopez-Rodriguez, Claire Gaveriaux-Ruff, John N. Wood

**Affiliations:** 1 Molecular Nociception Group, WIBR, University College London, Gower Street, WC1E 6BT, UK; 2 Immunology Unit, Department of Experimental and Health Sciences, Universitat Pompeu Fabra, Carrer Doctor Aiguader No88, 08003 Barcelona, Spain; 3 Institut de Génétique et de Biologie Moléculaire et Cellulaire, Université de Strasbourg, Centre National de la Recherche Scientifique , UMR7104, INSERM U1258, Ecole Supérieure de Biotechnologie de Strasbourg, Ilkirch, Strasbourg, France

**Keywords:** Nav1.7 channel, opioid receptors, pain, analgesia, behaviour

## Abstract

**Background: **Functional deletion of the *Scn9a* (sodium voltage-gated channel alpha subunit 9) gene encoding sodium channel Nav1.7 makes humans and mice pain-free. Opioid signalling contributes to this analgesic state. We have used pharmacological and genetic approaches to identify the opioid receptors involved in this form of analgesia. We also examined the regulation of proenkephalin expression by the transcription factor Nfat5 that binds upstream of the *Penk* gene.

**Methods: **We used specific µ-, δ- and κ-opioid receptor antagonists alone or in combination to examine which opioid receptors were necessary for Nav1.7 loss-associated analgesia in mouse behavioural assays of thermal pain. We also used µ- and δ-opioid receptor null mutant mice alone and in combination in behavioural assays to examine the role of these receptors in *Nav1.7* knockouts pain free phenotype. Finally, we examined the levels of *Penk* mRNA in *Nfat5*-null mutant mice, as this transcription factor binds to consensus sequences upstream of the *Penk* gene.

**Results:** The pharmacological block or deletion of both µ- and δ-opioid receptors was required to abolish *Nav1.7*-null opioid-related analgesia. κ-opioid receptor antagonists were without effect. Enkephalins encoded by the *Penk *gene are upregulated in *Nav1.7* nulls. Deleting *Nfat5*, a transcription factor with binding motifs upstream of *Penk*, induces the same level of enkephalin mRNA expression as found in *Nav1* *.7* nulls, but without consequent analgesia. These data confirm that a combination of events linked to *Scn9a* gene loss is required for analgesia. Higher levels of endogenous enkephalins, potentiated opioid receptors, diminished electrical excitability and loss of neurotransmitter release together contribute to the analgesic phenotype found in *Nav1.7*-null mouse and human mutants.

**Conclusions:** These observations help explain the failure of Nav1.7 channel blockers alone to produce analgesia and suggest new routes for analgesic drug development.

## Introduction

Pain is numerically the greatest clinical challenge of the age, affecting about half the population, whilst 7% of people have debilitating pain conditions ^1^. Finding new analgesic targets and drugs has proved challenging. One approach has been to identify the genes involved in human monogenic loss of pain conditions ^2^. The association of human gain-of-function mutations in Nav1.7 with enhanced pain phenotypes, and the pain-free state linked to loss of Nav1.7 expression focused considerable attention on this voltage-gated sodium channel as a potential analgesic drug target ^3^. Nav1.7 is found in damage-sensing peripheral sensory neurons, sympathetic neurons and CNS structures like the hypothalamus, as well as in non-neuronal locations such as the pancreas. Deletion in all sensory neurons and sympathetic neurons abolishes acute, inflammatory and neuropathic pain, although some pain disorders such as oxaliplatin-evoked cold allodynia are retained ^4, 5^.

As human and mouse *Nav1.7*-null mutants are effectively pain-free, this channel should be an excellent analgesic drug target ^6^. However, channel blockers are very weak analgesics ^3, 7^. This is likely due to the fact that partial channel blocking cannot recapitulate the many physiological effects of gene deletion. This explanation is supported by experiments that show that only 100% channel block with very high dose tetrodotoxin can recapitulate some effects of gene deletion ^8^. In null mutants, neurotransmitter release is diminished, and synaptic integration is also diminished. In addition, the opioid peptide enkephalins are upregulated in the absence of Nav1.7, and opioid receptor signalling is potentiated. Both of these latter events may be linked to loss of sodium ingress through Nav1.7 ^8^.

Consistent with an opioid component of analgesia, the opioid antagonist naloxone substantially reverses *Nav1.7* loss-associated pain free phenotype ^8^. We wondered which opioid receptors were involved in this process. Here, using pharmacological studies and opioid receptor knockout mice, we show that both µ-opioid receptors (MORs) and δ-opioid receptors (DORs) contribute to *Nav1.7*-null mutant analgesia and deleting both receptors mimics the effects of naloxone on *Nav1.7*-null analgesia in mice. In addition, we show that elevating enkephalin mRNA levels in NFAT5 null mutant mice similar to those found in *Nav1.7* nulls is not alone sufficient to cause measurable analgesia.

## Methods

### Animals

*Nav1.7* floxed mice were generated as described ^9^. Specific deletion of *Scn9a* exons 14 and 15 was performed by crossing *Nav1.7* ^flox/flox^ mice with *Wnt1-Cre* ^tg/0^ hemizygous transgenic mice purchased from Jackson Labs (129S4.Cg-Tg(Wnt1-cre)2Sor/J, Stock No: 022137). F1 offspring were crossed to obtain *Nav1.7* ^flox/flox^: *Wnt1-Cre* ^tg/0^ and further bred with either *MOR* ^-/-^ or *DOR* ^-/-^ mice. Previously reported *MOR*- and *DOR*-null mutants were used ^10, 11^. We obtained *Nav1.7* ^flox/flox^: *MOR* ^-/-^: *Wnt1-Cre* ^tg/0^ and *Nav1.7* ^flox/flox^: *DOR* ^-/-^: *Wnt1-Cre* ^tg/0.^ Finally, triple mutants carrying either *MOR* or *DOR* homozygous deletions were crossed in order to generate *Nav1.7* ^flox/flox^: *MOR* ^-/-^: *DOR* ^-/-^: *Wnt1-Cre* ^tg/0^. For all mouse lines, homozygous mutants were compared to Wnt1-Cre-negative animals. For clarity, *Nav1.7* ^flox/flox^: *DOR* ^+/+^: *Wnt1-Cre* ^0/0^ are named in this article Nav1.7 WT / DOR WT; *Nav1.7* ^flox/flox^: *DOR* ^+/+^: *Wnt1-Cre* ^tg/0^, Nav1.7 KO / DOR WT; *Nav1.7* ^flox/flox^: *DOR* ^-/-^: *Wnt1-Cre* ^0/0^, Nav1.7 wt / DOR KO; and finally *Nav1.7* ^flox/flox^: *DOR* ^-/-^: *Wnt1-Cre* ^tg/0^, Nav1.7 KO / DOR KO. The same simplification was applied for all the genotypes. *Nfat5* floxed mice were generated by Dr Cristina López-Rodriguez (Barcelona, Spain) ^12^.

Experiments were conducted using both male and female mice, which were between 8 and 12 weeks old at the time of experiments. Animals were housed up to five per cage, in a temperature-controlled room with a 12-h light–dark cycle. Food and water were available *ad libitum*. Genotyping was carried out on genomic DNA extracted from ear notches and PCR was conducted as described ^9– 11^. Mice were euthanized by gradual-fill CO _2_ gas followed by cervical dislocation at the end of experiments. A tail sample was further collected to confirm the genotype. Sample size for each experiment was established according to the literature. A total of 143 animals were used for the present work.

### Behavioural testing

Animal experiments were approved by the UK Home Office and UCL ethics committee Act 1986 with prior approval under a Home Office project licence (PPL 70/7382). Mice were acclimatized to the experimental room and were handled during a period of 1 week before starting the experiments. Observers who performed behavioural experiments were blinded to the genotype. All behaviour experiments were conducted between 14h and 18h. For the Hargreaves thermal test, the animal's hindpaw was exposed to an intense light beam and the withdrawal latency recorded manually using the Hargreaves’ apparatus (Ugo Basile) ^13^. For the Randall Selitto test, a blunt probe was used to apply force approximately midway along the tail (Ugo Basile) ^14^. For the hot plate test, animals were exposed to a 55°C chamber floor and the withdrawal latency recorded ^15^.

### Drugs

*In vivo experiments*. Naloxone, Naltrindole hydrochloride (NTI), CTOP and nor-Binaltorphimine dihydrochloride (norBNI) were purchased from Sigma, UK and dissolved in saline; they were respectively administered 30 min, 30 min, 15 min and 60 min, before performing behavioural experiments. Unless specified, all drugs were injected intraperitoneally at the dose described in the figure legend (typically, 2 mg/kg for naloxone, 5 mg/kg for NTI, 1.5 mg/kg for CTOP and 10 mg/kg for norBNI).

*In vitro experiments.* Monensin, TTX and Veratridine (Sigma, UK) were respectively dissolved in ethanol, saline and DMSO. Ionomycin (Molecular Probes) was resuspended in DMSO. Monensin at 500 nM was incubated with DRG neurons for 30 or 60 min. TTX at 500 nM and Veratridine at 1 µM were incubated 6h before harvesting the cells. For controls, the same volumes of vehicle were used. Same concentration of Monensin, TXT and Veratridine were applied in live cell imaging experiments, ionomycin was used at 200 nM.

### DRG neuron cultures

DRG from all spinal levels were harvested and dissociated as described ^16^. Dissociated neurons were plated on poly-L-lysine- and laminin-coated 35-mm plastic dishes (Nunc, Denmark). Incubation with drugs was started at least 24 h after dissociation. Monensin (Sigma, UK, in 100% ethanol), TTX (Sigma, UK, in extracellular solution) or Veratridine (Sigma, UK, DMSO) were used at concentrations described in the figure legends before RNA extraction and quantification. For each experiment, control DRG neurons were treated with the appropriate vehicle.

### Quantitative PCR

For fresh DRG analysis, DRG from lumbar segments L4, L5 and L6 were harvested and pooled. For DRG cultures, cells were collected after incubation with the drug and concentrated by centrifugation. RNA was extracted using TRIzol® Reagent (Invitrogen) according to the manufacturer’s instructions. Reverse transcription was performed using iScript™ Reverse Transcription Supermix (Bio-Rad) for RT-qPCR following the Bio-Rad supplied protocol. cDNA amplification was performed in triplicate, using SsoAdvanced™ Universal SYBR® Green Supermix (Bio-Rad) with the following primers; *Penk:* forward 5’ TTCAGCAGATCGGAGGAGT 3’, reverse 5’ AGAAGCGAACGGAGGAGAC 3’; *Nav1.7* ex 7 forward 5’ TTTCCGGAAGGACCTTGAGC 3’, reverse CTGCCCTGAATCTGTGCTGA; *Nav1.7* ex 14 forward 5’ GAGCACCATCCAATGACGGA 3’, reverse 5’ TTCAGCTGCGAAGATCCCTG 3’; *Nfat5* ex 3-4 forward 5’ AGTCAGACAAGCGGTGGTGA 3’, reverse 5’ CAGACACTCCCTGCTTCAGAG 3’; *Nfat5* ex 6-7 forward 5’ TTGCAGACACCTTCTTCCCC 3’, reverse 5’ CTCTCCTTTCACTGAACAGCTA 3’; *Gapdh* forward 5’ TGCGACTTCAACAGCAACTC 3’, reverse 5’ CTTGCTCAGTGTCCTTGCTG 3’. Amplification were conducted with the following program: 3 min at 95°C, 40 cycles of 60°C for 10 sec, 72°C for 10 sec, 95°C for 10 sec, and finally a melting curve for 10 min from 66°C to 100°C.

DNA amplification was quantified with a Bio-Rad CFX Connect™ Real-Time PCR Detection System thermocycler. The expression level of target genes was normalized to housekeeping gene mRNA ( *Gapdh*). Fold changes were determined using the 2 ^−ΔΔCt^ equation ^17^, in which wild-type littermate or vehicle-treated cultured DRG cDNA samples were designated as the calibrator. The data presented are given as the mean of the fold changes.

### Live cell imaging

For Na ^+^ imaging, neurons were loaded for 30 min with 5 µM of SBFI in serum free DMEM, and then washed with extracellular solution (140 mM NaCl, 3 mM KCl, 10 mM HEPES, 10 mM D-Glucose, 2 mM CaCl _2_, 1 mM MgCl _2_, pH 7.4 adjusted with KOH, Osmolarity 300 mOsm adjusted with D-Glucose). Cells were alternately excited at 340 and 380 nm and emissions at 510 nm collected separately to determine 340/380 nm ratio. Calibration of [Na ^+^] _i_ was performed by exposing SBFI-loaded DRG neurons to different extracellular solutions with specific Na ^+^ concentration for 30 min (in the additional presence of 3 µM gramicidin D for equilibrium between intracellular and extracellular Na+ concentration). For Ca ^2+^ imaging, cells were loaded with 1 µM of Fura-2 for 30 min and alternatively excited at 340 and 380 nm. Results were expressed using the ratio of the 340 nm/380 nm wavelengths.

### Statistical analysis

Data were analysed using GraphPad Prism 7 (GraphPad Software Inc., San Diego, CA) and presented as mean ± SEM. Statistically significant differences between two groups were assessed by two-tailed unpaired t-test. p<0.05 was considered significant. Statistically significant differences between more than two groups were assessed by one-way ANOVA or two-way ANOVA for respectively non-repeated and repeated measures, followed by the post hoc test indicated in the figure legend. p<0.05 was considered significant. Statistical tests performed for a given experiment are described in figure legends.

An earlier version of this article can be found on bioRxiv (DOI: https://doi.org/10.1101/297184).

## Results

### Effects of MOR deletion on pain perception

**Figure 1.**
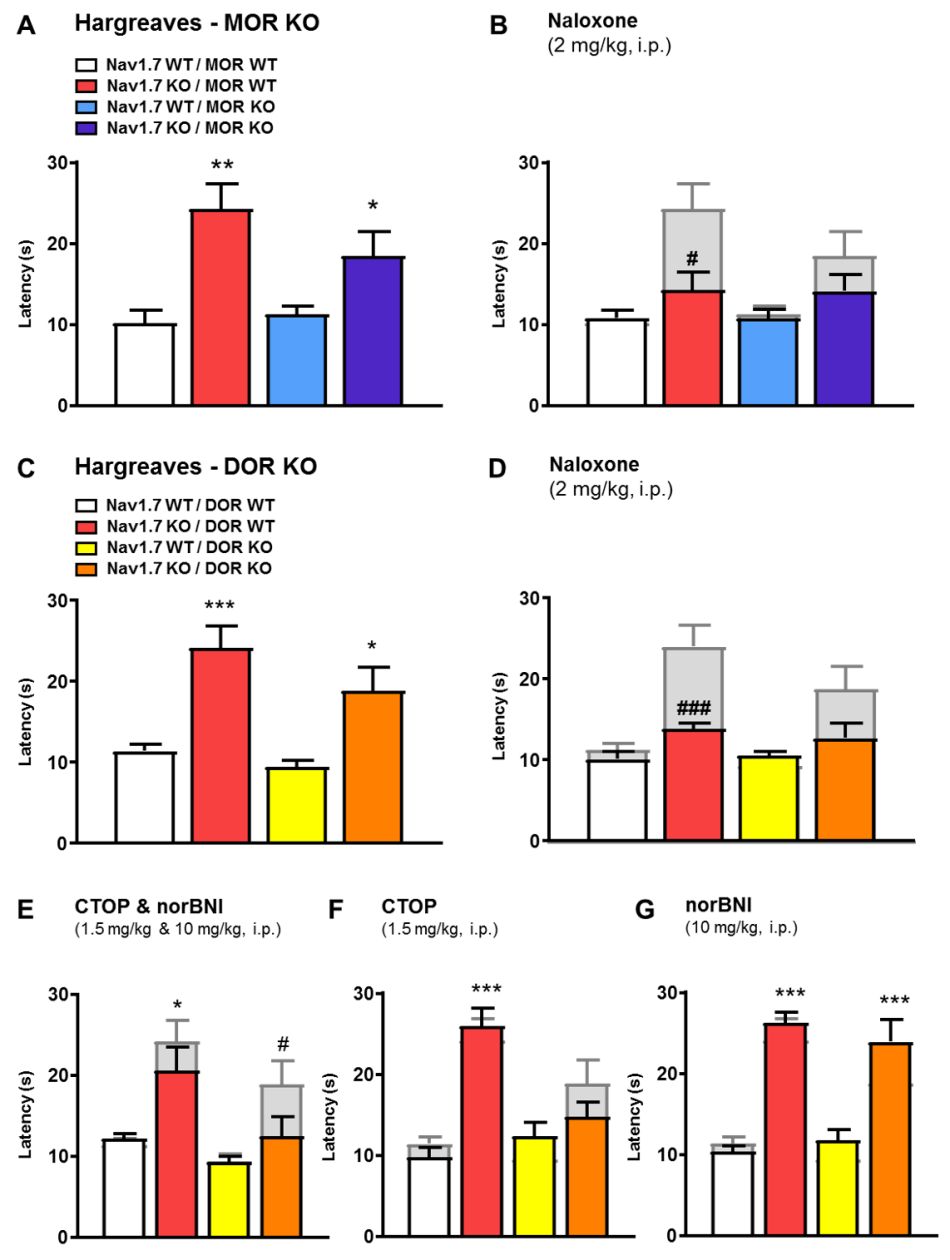
µ-opioid receptor (MOR) or δ-opioid receptor (DOR) deletion is not sufficient to reduce *Nav1.7* knockout (KO) pain sensitivity. ( **A**) Noxious thermal stimulation of Nav1.7 WT/MOR WT (white), Nav1.7 KO/MOR WT (red), Nav1.7 WT/MOR KO (blue) and Nav1.7 KO/MOR KO (purple) mouse hindpaw using Hargreave's apparatus (n=5–9 per group). ( **B**) Hindpaw withdrawal latency 20 min after naloxone administration (2 mg/kg, i.p). The grey bars represent noxious thermal withdrawal latency baselines merged with latency measured 20 min after naloxone to facilitate comparison between pre and post drug injection pain-related behaviour. The same representation of baselines by grey bars has been applied for all behavioural experiments. Results are presented as means ± SEM. Data were analysed by one-way ANOVA followed by Dunnett's post hoc test ( **A**) or two-way ANOVA followed by Bonferroni post hoc test ( **B**). * p<0.05 ** p<0.01 vs Nav1.7 WT/MOR WT; # p<0.05 vs own baseline). ( **C**) Noxious thermal stimulation of Nav1.7 WT/DOR WT (white), Nav1.7 KO/DOR WT (red), Nav1.7 WT/DOR KO (yellow) and Nav1.7 KO/DOR KO (orange) mice (n=8 per group). ( **D**) Hindpaw withdrawal latency 20 min after naloxone administration (2 mg/kg, i.p.). ( **E**) Thermal withdrawal latency after a combination of the MOR antagonist CTOP (1.5 mg/kg, i.p.) and the kappa antagonist norbinaltorphimine (norBNI) (10 mg/kg, i.p.,) injected respectively 15 and 60 min before the test. ( **F**) Effect of CTOP and ( **G**) norBNI on mouse hindpaw withdrawal latency using Hargreave's test (administrated 15 min or 60 min before recording the latency). Results are presented as mean ± SEM. Data were analysed by one-way ANOVA followed by Dunnett's post hoc test ( **C**) or two-way ANOVA followed by the Bonferroni post hoc test ( **D**– **G**). * p<0.05 ** p<0.01 and *** p<0.001 vs Nav1.7 WT / DOR WT, # p<0.05 ## p<0.01 and ### p<0.001 vs own baseline.

We first examined the role of MORs in *Nav1.7*-null-associated analgesia ( Figure 1A, B). *Nav1.7*-null mutant mice show dramatic thermal analgesia. Global deletion of *MOR* on a *Nav1.7*-null background had a small effect on acute heat pain behaviour ( Figure 1A). This effect did not match the effects of naloxone, which substantially diminished analgesia ( Figure 1B). Consistent with this, naloxone further diminished the analgesic phenotype of *Nav1.7*/ *MOR* double-mutant mice, demonstrating that MORs alone do not account for the opioid-mediated component of Nav1.7-null-associated analgesia ( Figure 1B).

### Effects of DOR deletion on pain perception

Next, we tested the effect of deleting *DOR* on *Nav1.7*-null pain behaviour ^10^. Once again, there was a small diminution in analgesia compared to *Nav1.7*-null mice ( Figure 1C). Naloxone further diminished the analgesic phenotype of the *Nav1.7*/ *DOR* double-null mutants ( Figure 1D), demonstrating that DORs alone do not account for the opioid-mediated component of *Nav1.7*-null-associated analgesia. However, when the potent selective MOR antagonist CTOP was applied to DOR receptor-null mice ^18^, the analgesia associated with Nav1.7 deletion was reduced by the same level as with naloxone ( Figure 1F). CTOP and the κ-opioid receptor (KOR) antagonist norbinaltorphimine (norBNI) ^19^ together also had the same effect as naloxone when applied to a *Nav1.7*/ *DOR* double-null animal ( Figure 1E). However, norBNI on a *Nav1.7*/ *DOR* null background was without effect ( Figure 1G). These data show that KORs do not mediate analgesia in *Nav1.7*-null mutants, but pharmacological block of MOR on a *DOR*-null background can account for all opioid-mediated analgesia.

### Effects of double MOR/DOR deletion on pain perception

**Figure 2.**
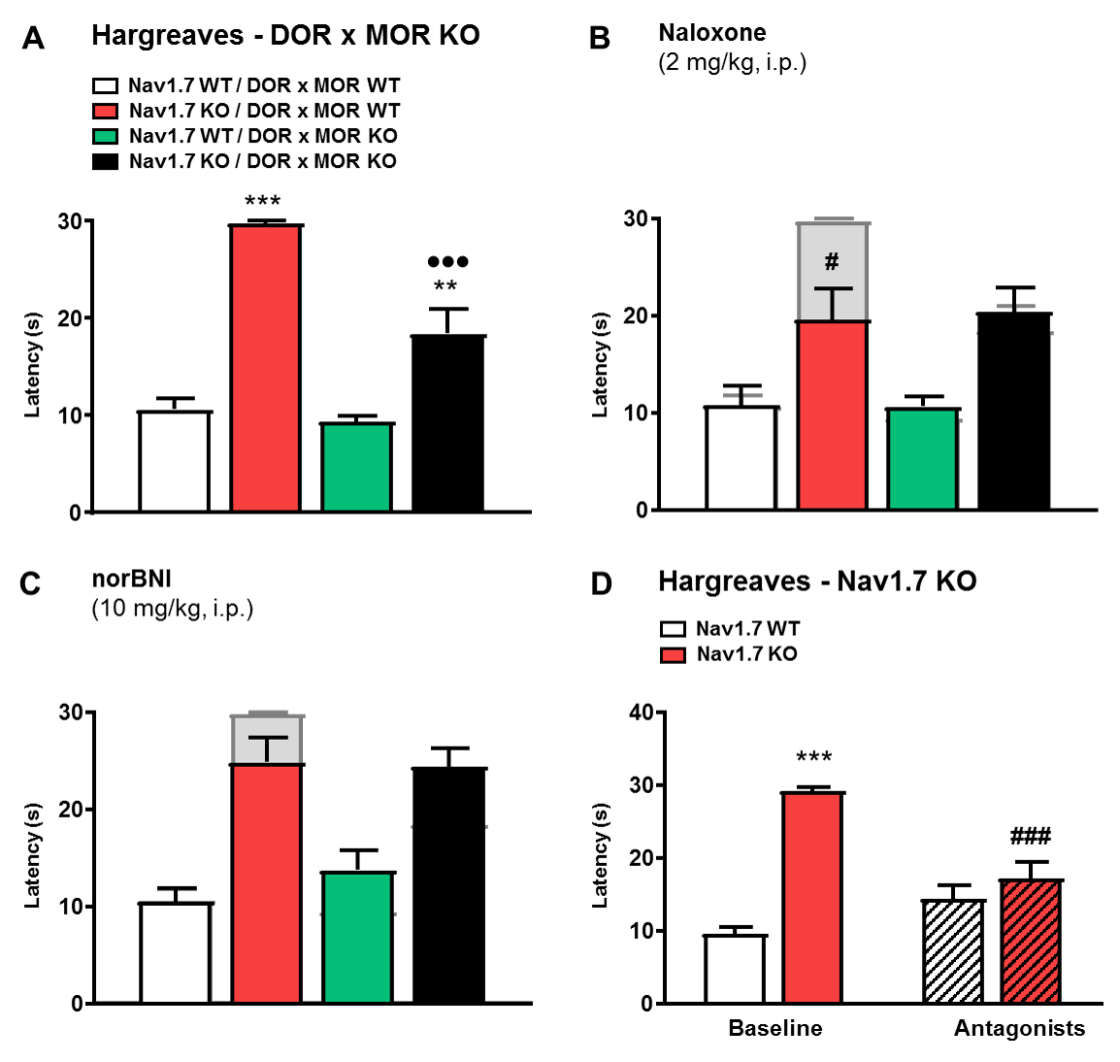
Deletion of both MOR and DOR mimics Naloxone effects on Nav1.7 knockout (KO) pain thresholds. ( **A**) Noxious thermal stimulation of Nav1.7 wild type (WT)/DOR x MOR WT (white), Nav1.7 KO/DOR x MOR WT (red), Nav1.7 WT/DOR x MOR KO (green) and Nav1.7 KO/DOR x MOR KO (black) mice hindpaw using Hargreave's apparatus (n=7–8 per group). ( **B**) Hindpaw withdrawal latency 20 min after naloxone administration (2 mg/kg, i.p., saline). The grey bars represent noxious thermal withdrawal latency baselines merged with latency measured 20 min after naloxone to facilitate comparison between pre and post drug injection pain-related behaviour. ( **C**) Thermal withdrawal latency after administration of norbinaltorphimine (norBNI) (10 mg/kg, i.p.) injected 60 min before the test. Results are presented as mean ± SEM. No statistically significant effect was seen. Data were analysed by one-way ANOVA followed by Dunnett's post hoc test ( **A**) or two-way ANOVA followed by the Bonferroni post hoc test ( **B** and **C**). ** p<0.01 and *** p<0.001 vs Nav1.7 WT/DOR x MOR WT; # p<0.05 vs own baseline; ••• p<0.001 vs Nav1.7 WT/DOR x MOR KO. ( **D**) Hindpaw withdrawal latency after administration of a combination of CTOP (2 mg/kg, i.p., saline, injected 15 min before the test), NTI (5 mg/kg, s.c., 30 min before test) and norBNI (10 mg/kg, i.p. 60 min before test) in WT (white bars) or Nav1.7 KO mice (red bars). Co-injection of MOR, DOR and κ-opioid receptor antagonists restores Nav1.7 KO thermal sensitivity. Results are presented as mean ± SEM. Data were analysed by two-way ANOVA followed by the Bonferroni post hoc test. *** p<0.001 vs Nav1.7 WT; ### p<0.001 vs baseline.

To provide further evidence that both MOR and DOR contribute to opioid-mediated analgesia in *Nav1.7* nulls, we generated double opioid receptor null mutant mice on a *Nav1.7* null background. Double *MOR*/ *DOR* knockouts on a *Nav1.7*-null background showed exactly the same loss of analgesia as that caused by naloxone in *Nav1.7* knockout mice ( Figure 2A, B). Application of MOR, DOR and KOR antagonists ^20^ together did the same ( Figure 2D), although the KOR antagonist norBNI alone showed no statistically significant effect, confirming that KOR activation did not contribute to analgesia ( Figure 2C). These pharmacological and genetic studies demonstrate that MOR and DOR together account for opioid-mediated analgesia in *Nav1.7*-null mutant mice.

### Assessing the effect of sodium levels on *Nav1.7* and *Nfat5* transcription

**Figure 3.**
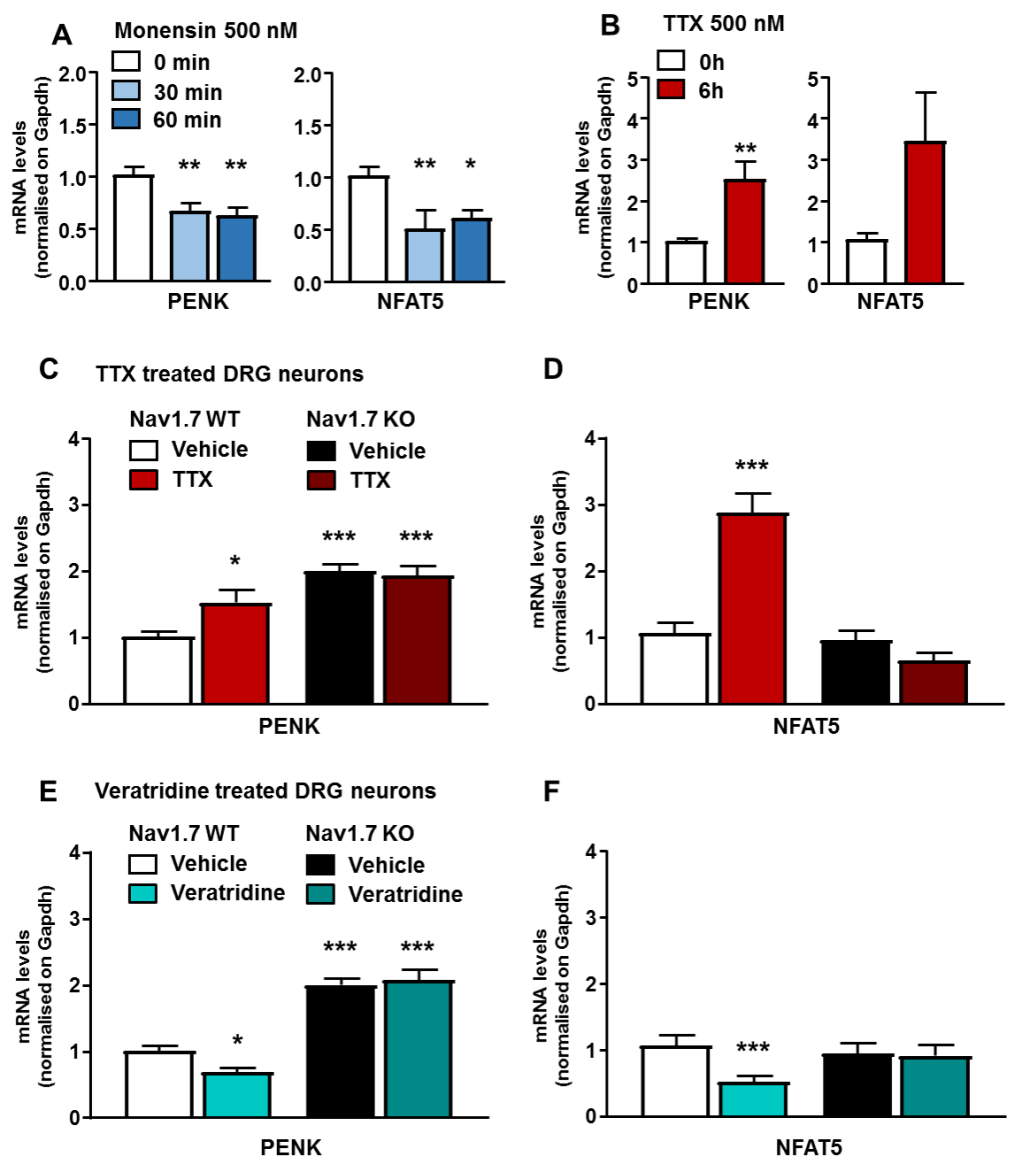
Both *Penk* and *Nfat5* expression are regulated by intracellular sodium concentration. ( **A**) *Penk* and *Nfat5* expression levels in cultured DRG neurons treated with monensin (500 nM, 30 and 60 min, respectively, light and dark blue bars). Control neurons (white bar) were treated with vehicle (ethanol) for 60 min. ( **B**) *Penk* and *Nfat5* mRNA quantification in cultured DRG neurons treated with tetrodotoxin (TTX) (500 nM, 6 h). Control neurons received same volume of saline solution for 6 h (red bar). ( **C**) *Penk* and ( **D**) *Nfat5* transcripts levels in wild-type (WT) compared to Nav1.7 knockout (KO) DRG neurons treated by TTX (500 nM, 6h). TTX induced *Penk* overexpression is correlated with *Nfat5* expression level, both are dependant of Nav1.7. ( **E**) *Penk* and ( **F**) *Nfat5* expression in WT and Nav1.7 KO cultured DRG neurons treated with veratridine (1 µM, 6h). Results are presented as mean ± SEM. Data were analysed by two-way ANOVA followed by the Bonferroni post hoc test. * p<0.05 ** p<0.01 and *** p<0.001 vs Nav1.7 WT Vehicle.

Elevated levels of enkephalins are found in *Nav1.7*-null mutant mice ^8^. Notably, there are five consensus binding sites for the transcription factor Nfat5 upstream of the *Penk* coding region. Nfat5 recognizes DNA elements similar to those bound by Nfatc proteins ^21^. As Nfat5 activity is regulated by hyper-osmolarity and salt kinases ^22^, there is a potential link between sodium ingress through Nav1.7 and transcriptional regulation. We manipulated sodium levels in sensory neuron cultures using either monensin as a sodium ionophore (control [Na ^+^] 6.65 mM, SEM 0.27; [Na ^+^] monensin 9.46 mM, SEM 0.44; n = 19;) or veratridine as an activator of voltage-gated sodium channels (control [Na ^+^] 5.5 mM, SEM 0.25; [Na ^+^] veratridine 7.6 mM, SEM 0.41; n = 9) to increase sodium levels, and very high doses of tetrodotoxin (TTX) (500 nM) to block voltage-gated sodium channel activity and potentially lower intracellular sodium ( Supplementary File 1). Notably, agents that alter intracellular sodium concentrations impact similarly on *Nav1.7* and *Nfat5* mRNA levels. Monensin ( Figure 3A) lowered both *Penk* and *Nfat5* mRNA levels, whilst TTX elevated them ( Figure 3B). The TTX effect was apparent in wild-type mice, but not in *Nav1.7* nulls, implying that this channel is the locus of action for *Penk* mRNA control by TTX ( Figure 3C, D).

### Effect of Nav1.7/Nfat5 knockout on pain reception

**Figure 4.**
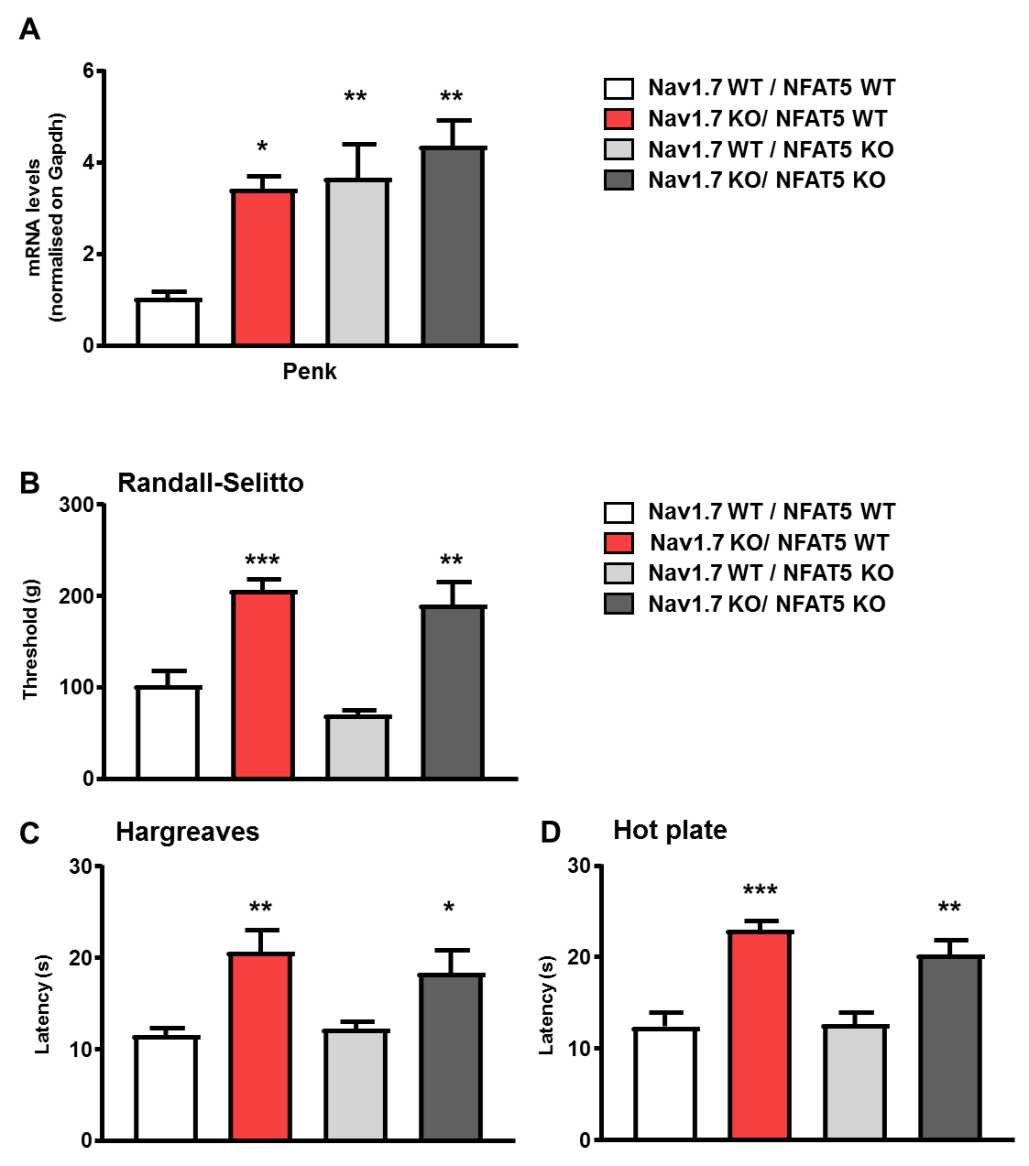
*Nfat5* conditional gene deletion induces *Penk* overexpression *in vivo* without eliciting a pain-insensitive phenotype. ( **A**) Expression levels of *Penk* transcript in Nav1.7 WT/NFAT5 wild type (WT) (white bar), Nav1.7 knockout (KO)/NFAT5 WT (red bar), Nav1.7 WT/NFAT5 KO (light grey) and Nav1.7 KO/NFAT5 KO mice (dark grey). ( **B**) Noxious mechanical pressure threshold of the same four mice lines using the Randall-Selitto apparatus. ( **C**) Noxious thermal stimulation of Nav1.7 WT/NFAT5 WT (white bar), Nav1.7 KO/NFAT5 WT(red bar), Nav1.7 WT/NFAT5 KO (light grey) and Nav1.7 KO/NFAT5 KO mice (dark grey) mice hindpaw using Hargreave's apparatus (n=7–10 per groups). ( **D**) Response to noxious thermal stimulation by using the hotplate test at 55°C. Results are presented as mean ± SEM. Data were analysed by one-way ANOVA followed by the Dunnett’s post hoc test. * p<0.05 ** p<0.01 and *** p<0.001 vs Nav1.7 WT/NFAT5 WT.

Veratridine lowered both *Penk* and *Nfat5* mRNA levels in wild-type, but not in *Nav1.7*-null mutant mice, again linking transcriptional events to Nav1.7 channel activity ( Figure 3E, F). We examined the role of *Nfat5* using conditional *Nfat5-Wnt1-Cre* null mutants in sensory neurons of wild-type and *Nav1.7*-null mutant mice. Expression levels of *Nfat5* and *Nav1.7* transcripts in single- and double-mutants were analysed to confirm Cre activity at the floxed loci ( Supplementary File 2). *Nfat5* conditional null mutant mice showed enhanced expression of *Penk* mRNA ( Figure 4A). When the *Nfat5*-null mice were crossed with *Nav1.7*-null mutants, *Penk* mRNA levels further increased ( Figure 4A). As *Nfat5*-null mice have the same levels of *Penk* mRNA as *Nav1.7*-null mutants, this allowed us to examine the contribution of enhanced opioid peptide expression to the analgesia seen in *Nav1.7*-null mutant mice. Opioid signalling in *Nav1.7*-null mutants is potentiated in at last two ways. First, there are enhanced levels of enkephalins, and second the opioid receptors have much enhanced activity, as measured indirectly through the quantitation of protein kinase A signalling ^23^. There was, perhaps surprisingly, no analgesic effect of elevated enkephalin levels in the *Nfat5-*null sensory ganglia. By measuring noxious mechanosensation ( Figure 4B), thermal thresholds ( Figure 4C), and noxious heat-induced-pain-related behaviour ( Figure 4D), the *Nfat5*-null enkephalin-induced mice showed normal pain behaviour, compared to *Nav1.7*-null mice ( Figure 4). As opioids clearly play a role in *Nav1.7*-null analgesia, as demonstrated by the naloxone effects, this suggests that the enhanced activity of opioid receptors may make a major contribution to *Nav1.7*-null opioid-mediated analgesia.

All raw data are available on OSF ^24^.

## Discussion

What are the implications of these findings for drug development? Firstly, the complexity of physiological changes that occur in *Nav1.7*-null mice is striking. Receptors (e.g. 5HTr4) and transcription factors (e.g. Runx1) implicated in nociception are dysregulated ^8^, opioid peptide expression is increased ^8^ and opioid signalling is potentiated ^23^, whilst electrical excitability ^25^ and integration of nociceptive stimuli is lost ^26^. There is evidence that these events require the complete loss of Nav1.7 function, as occurs in null mutants. For example, only complete channel blockade with very high doses of TTX can induce increased *Penk* mRNA expression ^8^. Should small-molecule-specific Nav1.7 antagonists be able to replicate all these events then they would be excellent analgesics. All the evidence thus far demonstrates that this is not the case, and the necessarily partial blockade of Nav1.7 does not cause analgesia ^7^. Molecules with limited specificity, like Biogen’s BIIB074, are good analgesics, but much of their activity likely results from blockade of sodium channels other than Nav1.7 ^22^.

The role of MOR and DOR and the lack of a role for KOR in *Nav1.7*-null analgesia fit with recent data. There is evidence for MOR–DOR interactions in nociceptive sensory neurons ^27^, and primates express MOR–DOR heteromultimers as targets of opioid analgesia ^28^. As *Nav1.7* deletion in peripheral nervous system-dependent Cre mice causes analgesia, then the actions on opioid receptors must occur either on primary sensory neurons, or on their synaptic targets within the spinal cord. Evidence that co-administration of opioids with Nav1.7 antagonists can have synergistic therapeutic effects has been demonstrated with a number of specific Nav1.7 antagonists. However, human proof-of-concept studies on synergistic analgesia with Nav1.7 antagonists and opioids have yet to be published. The evidence for potentiation of opioid receptor signalling in *Nav1.7*-null mice is significant ^23^. Although diminished electrical excitability may provide the necessary landscape for endogenous opioid effects, it is surprising that elevated enkephalin levels alone do not produce any detectable levels of analgesia in the *Nfat5*-null mice. Exogenous administration of enkephalins in humans delivered through gene therapy has useful analgesic effects ^29^. The focus is then upon potentiated opioid receptor signalling ^23^. There is some evidence linking the ingress of sodium through Nav1.7 to effects on G-protein-coupled receptor (GPCR) activity. Pert and Snyder showed the influence of sodium on opioid receptor activity in 1974, demonstrating that increased sodium concentrations caused diminished agonist binding ^30^. Intracellular sodium levels may control this process ^31^ and the proximity of Nav1.7 channels to opioid receptors may influence sodium occupancy of these GPCRs ^32^.

In summary, MORs and DORs are required for the opioid component of *Nav1.7*-null mutant analgesia. Co administration of MOR/DOR agonists with specific Nav1.7 antagonists may therefore have useful analgesic effects ^33^. If analgesia depends substantially upon both potentiated receptor activity, as well as increased enkephalin expression, analgesic drug development using small molecules to mimic *Nav1.7* gene deletion will be problematic. Nociceptor silencing through CRISPR-mediated gene deletion of *Nav1.7* may prove a more tractable analgesic strategy for extreme chronic pain conditions ^34^.

## Data availability

Raw values in GraphPad Prism files for behavior and expression analysis and raw data for live imaging in Excel file are deposited in OSF: https://dx.doi.org/10.17605/OSF.IO/HWZ6E ^24^.

## Supplementary material

**Supplementary File 1. Live imaging in DRG neurons of sodium and calcium levels.** Live recording of intracellular sodium or calcium variations in DRG neurons in culture upon exposure to either Monensin, TTX, Veratridin or Ionomycin.

**Supplementary File 2. mRNA levels in double *Nav1.7**Nfat5* KO.** Confirmation of mice genotype by qPCR.
