# Supplementary material for: Analgesia linked to Nav1.7 loss of function requires µ- and δ-opioid receptors
Source: Wellcome Open Res. 2018 Aug 16;3:101. [Version 1] doi: 10.12688/wellcomeopenres.14687.1 (PMC6134336; doi:10.12688/wellcomeopenres.14687.1)
Supplement: Supplementary file 1 [file wellcomeopenres-3-15991-s0000.tgz › 2562784a-9428-4bfe-b439-4f3a4d0df570.docx]

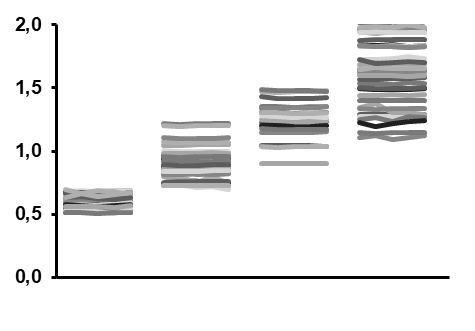


**Ratio 340/380**

**Na^+^ Free**

**Na^+^ 5 mM**

**Na^+^ 10 mM**

**Na^+^ 15 mM**


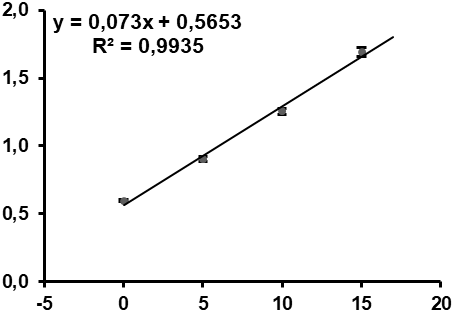


**Ratio 340/380**

**[Na^+^] (mM)**

**A**

**B**

**Supplementary data**

**Supplementary data:** (**A**) SBFI ratio 340/380 in function of intracellular sodium concentration. Intracellular concentration of DRG neurons was fixed by perfusing for 30 min extracellular solution of known Na+ concentration complemented by granamycin and monensin to allow an equilibrium between extracellular and intracellular concentration of Na+. (**B**) Corresponding calibration curve used to convert SBFI ration in sodium concentration.


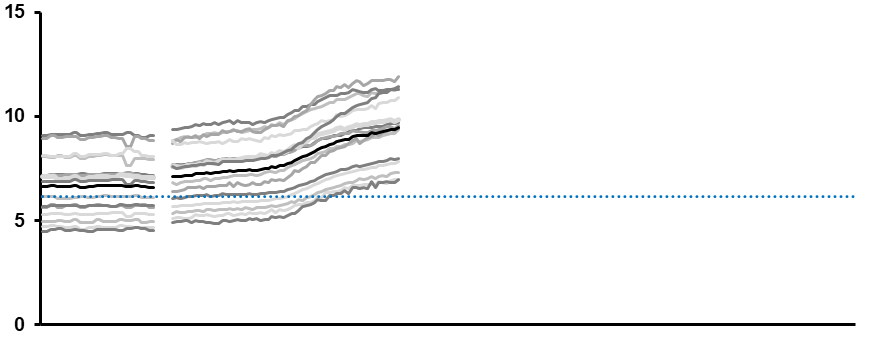


**Monensin 500 nM**

**20 min**

**30 sec**

**[Na+] (mM)**

**SBFI**


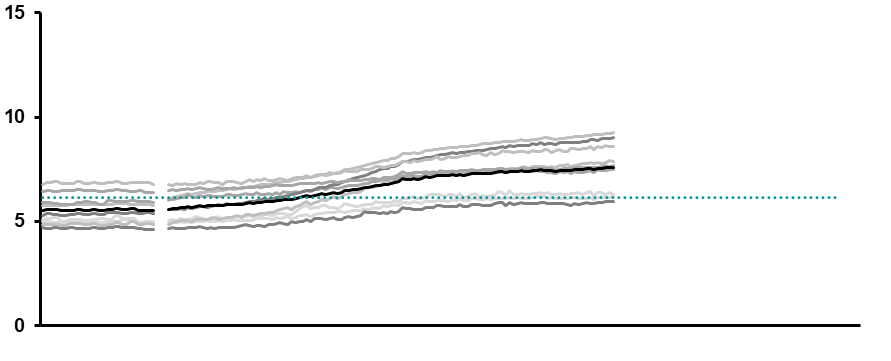


**Veratridine 1 µM**

**40 min**

**30 sec**

**[Na+] (mM)**


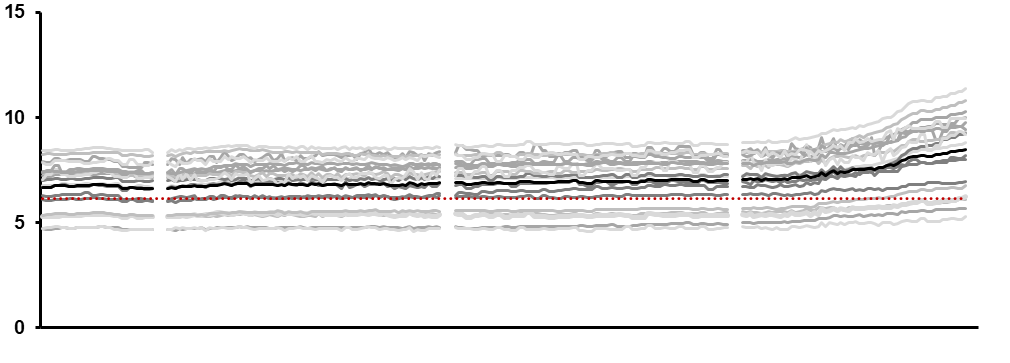


**TTX 500 nM**

**Veratridine 1 µM**

**Monensin 500 nM**


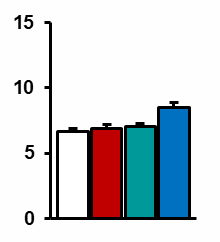


**25 min**

**25 min**

**30 sec**

***

**[Na+] (mM)**

***

**[Na+] (mM)**


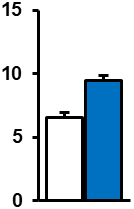

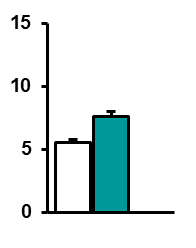


***

**[Na+] (mM)**

**A**

**B**

**C**

**Supplementary data:** (**A**) Monensin perfusion induces an increase in sodium intracellular concentration in cultured DRG neurons. This increase is significantly different when the 30 sec of baseline are compared to the last 30 sec recorded (right panel, white bar for baseline, blue bar for Monensin 500 nM). (**B**) Veratridine exposure induces an augmentation of intracellular sodium concentration. Histograms correspond to the first and last 30 sec of the experiment. (**C**) TTX perfusion do not change the sodium concentration but blocks veratridine effect. Histograms correspond to the first and last 30 sec of the experiment.


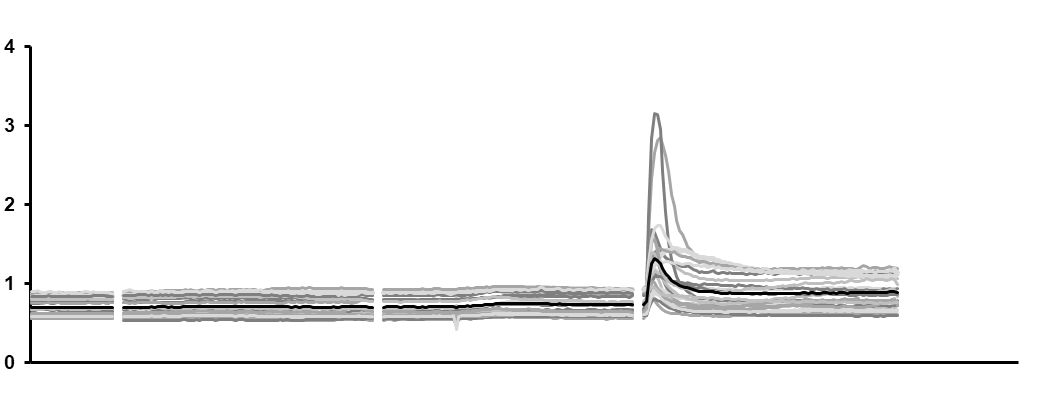

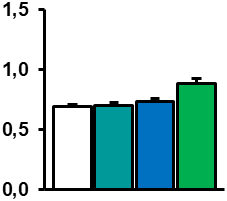


***

**Veratridine 1 µM**

**Monensin 500 nM**

**Ionomycin 200 nM**

**30 min**

**30 sec**

**[Ca^2+^] (Ratio 380/340 nm)**


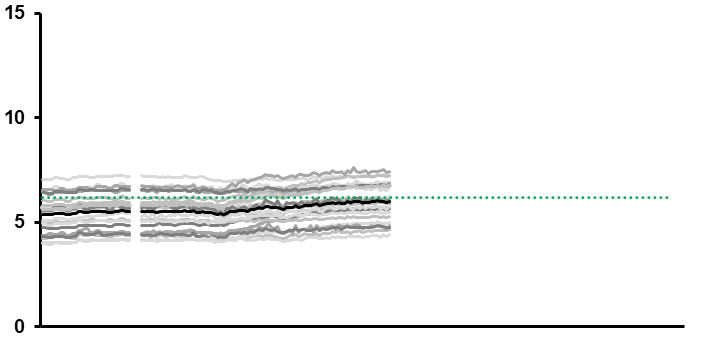


**Ionomycin 200 nM**

**30 min**

**30 sec**

**[Na+] (mM)**


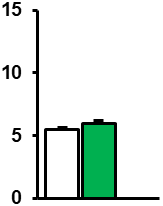


**[Na+] (mM)**

**A**

**B**

**Supplementary data:** (**A**) Neither Veratridine nor Monensin alter calcium concentrations in cultured DRG neurons. Ionomycin was used as a positive control triggering calcium concentration rise. (**B**) Ionomycin does not Impair Na+ intracellular concentration. Histograms correspond to the first and last 30 sec of the experiment for each perfused drug.
