# Supplementary material for: Analgesia linked to Nav1.7 loss of function requires µ- and δ-opioid receptors
Source: Wellcome Open Res. 2018 Aug 16;3:101. [Version 1] doi: 10.12688/wellcomeopenres.14687.1 (PMC6134336; doi:10.12688/wellcomeopenres.14687.1)
Supplement: Supplementary file 2 [file wellcomeopenres-3-15991-s0001.tgz › a13f23c4-1244-48b0-a210-d40d53741f4e.docx]

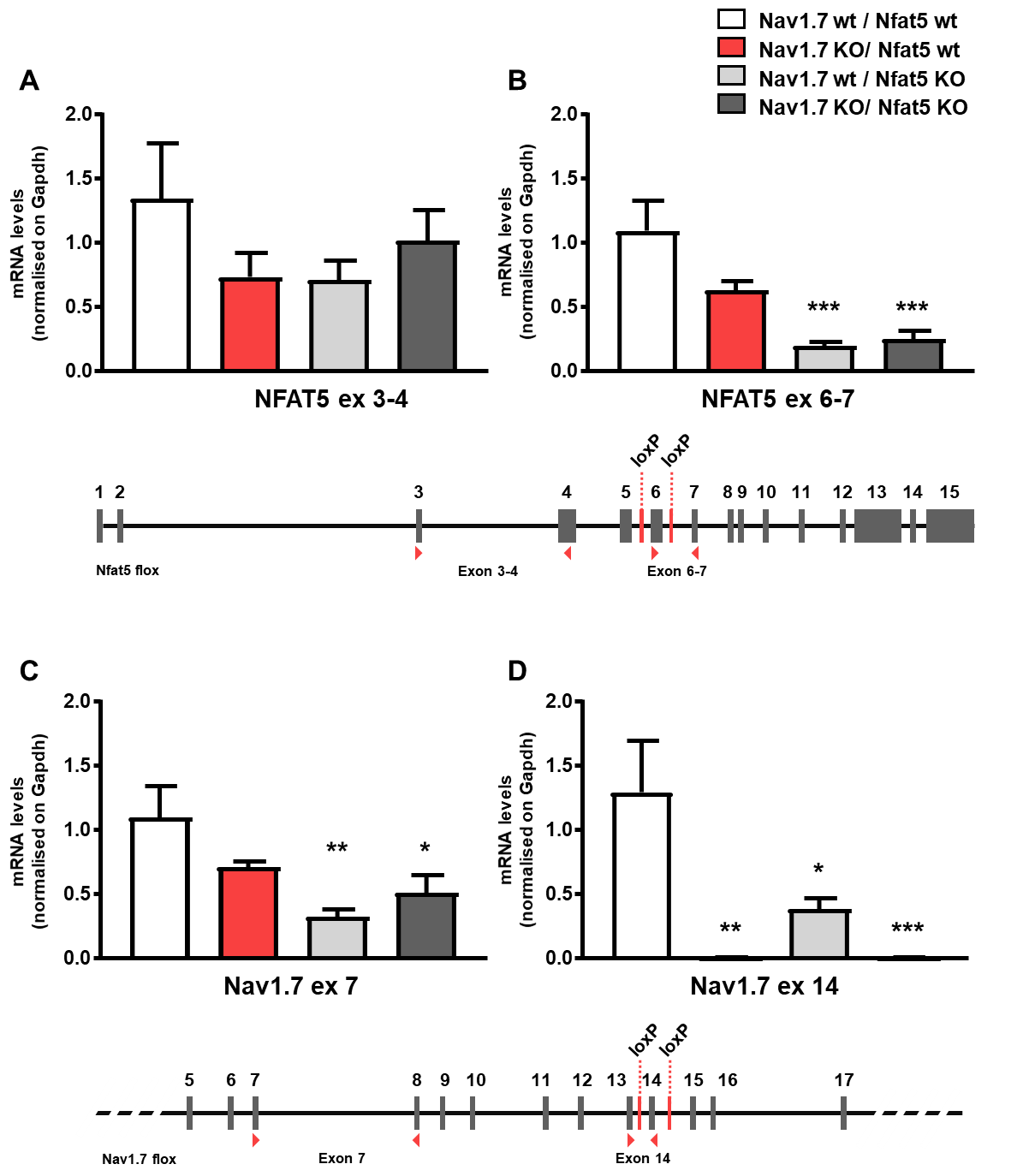


**Supplementary data** Expression levels of (**A**) NFAT5 total transcript, (**B**) NFAT5 full length transcript, (**C**) Nav1.7 total mRNA and (**D**) Nav1.7 full length transcript in Nav1.7 WT / NFAT5 WT, Nav1.7 KO / NFAT5 WT, Nav1.7 WT / NFAT5 KO and Nav1.7 KO / NFAT5 KO mice. Results are presented as mean ± SEM. Data were analysed by one-way ANOVA followed by the Dunnett’s post hoc test. * p<0.05 ** p<0.01 and *** p<0.001 vs Nav1.7 WT/ NFAT5 wt.
