# Supplementary material for: Analgesia linked to Nav1.7 loss of function requires µ- and δ-opioid receptors
Source: Wellcome Open Res. 2018 Aug 16;3:101. [Version 1] doi: 10.12688/wellcomeopenres.14687.1 (PMC6134336; doi:10.12688/wellcomeopenres.14687.1)
Supplement: Peer review history

# Peer review: Referee response for version 1

**DOI:** 10.21956/wellcomeopenres.15991.r33712

**Published:** 2018-09-11

**Authors:** Terrance P. Snutch

**Affiliations:** 1 Michael Smith Laboratories, Djavad Mowafaghian Centre for Brain Health, University of British Columbia, Vancouver, BC, Canada

In their manuscript Pereira and coworkers nicely combine a Nav1.7 null mouse model with pharmacological, gene knock-out and behavioural analyses to probe the relationship between the well-described loss of pain phenotype associated with Nav1.7 loss-of-function (LOF) mutations and a previous observation linking the null phenotype to potentiated opioid receptor-mediated signalling. The Wood group has previously shown that Nav1.7 (but not Nav1.8) null animals exhibit enhanced encephalin expression and concomitant increased opioid receptor signalling mediated via changes in gene expression. Here, examining the involvement of specific opioid receptors by administration of subtype-specific antagonists they find evidence for the combined contribution of MORs and DORs. These data are nicely confirmed by genetically crossing Nav1.7 with MOR and DOR null mice strains. Any contribution of KORs is eliminated pharmacologically using the selective KOR antagonist, norBNI.

 The authors further examine putative transcriptional roles for Nav1.7 channel activity and Na ions by altering intracellular Na level in primary cultured DRGs via a sodium ionophore and Na channel modulators.

 The results suggest that increasing Na levels decreases the expression of both PENK and nFAT5 and that the underlying mechanism is Nav1.7-dependent. Interestingly, in examining an nFAT5 conditional knock-out the authors find that PENK mRNA levels are increased without affecting Nav1.7 pain phenotypes.

**Comments**

 Overall, the paper is very well written, the experiments follow a logical pathway, and the data are analyzed carefully and properly. The notion that Na channel activity and/or intracellular Na levels contribute towards the transcriptional regulation of a selected subset of pain pathway genes is quite intriguing and the results will likely be of significant interest in the field. In part, the authors also conclude that their results explain the current inability across academia and industry to generate efficacious Nav1.7 blockers towards the treatment of pain.

 Figure 3. For clarity please add to the legend the mouse strain utilized in experiments in Panels A and B.

 Figure 3. The data examining for the effects of TTX are not entirely clear in my view. Supp File-1, Fig. 2C appears to show that 500 nM TTX had no effect on intracellular Na levels yet the mRNA levels in DRGs for both PENK and nFAT5 are significantly increased by TTX (Fig. 3B). How do the authors explain this discrepancy?

 Figures 3 and 4. The authors only examine the expression of PENK and nFAT in their study. In any qPCR expression study it is always most convincing if data are presented wherein some sort of target/marker is not altered by the treatment(s) implemented. In this regard, do they have (or can provide) qPCR data showing some sort of DRG-expressed marker is unchanged by the various treatments and genetic backgrounds? Preferentially something related to pain signalling.

 Figure 4A legend. Please clearly indicate the tissue/sample utilized in the expression studies.

 What do the authors ultimately propose as the linkage between Nav1.7 channel activity (or lack-there-of in the null), PENK and nFAT5 levels to collectively mediate transcriptional regulation? A direct transcriptional effect driven by Na? Or do Na levels alter other signalling cascades? (in Supp File-1, Fig. 3 they rule out an effect of global change in Ca levels by veratridine and monensin). Further in this regard, can the authors expand on the fact that PENK levels do not appear linked to nFAT expression (Fig. 4).   

 The involvement of RNA Pol II towards the proposed Na-level mediated transcriptional changes (PENK, nFAT5) should properly be confirmed via application of any of the well-described inhibitors commercially available (e.g., alpha-amanitin, actinomycin-D, triptolide).

 Supplemental File 1: It would be helpful to the reader if the various figures are numbered (Fig. 1, 2, 3). Also, for each of three Figs. – for clarity please add into the legends the n’s for the number of DRGs tested under each of the conditions.

 Both male and female animals were utilized throughout. Can the authors please confirm that there were no sex-specific differences either within or across the various data sets?

 Abstract – Results: The authors state here: “Higher levels of endogenous enkephalins, potentiated opioid receptors, diminished electrical excitability and loss of neurotransmitter release together contribute to the analgesic phenotype found in *Nav1.7*-null mouse and human mutants.”

 The reader may be confused by the statement in the Abstract given that the data in the paper do not directly address the levels of endogenous enkaphalins, measurements of electrical activity or that for neurotransmitter release. Please reword this statement to better reflect the data presented.

I have read this submission. I believe that I have an appropriate level of expertise to confirm that it is of an acceptable scientific standard.

# Peer review: Referee response for version 1

**DOI:** 10.21956/wellcomeopenres.15991.r33711

**Published:** 2018-09-03

**Authors:** C. Geoffrey Woods

**Affiliations:** 1 Department of Medical Genetics, Cambridge Institute For Medical Research, Cambridge, UK

**Analgesia linked to Nav1.7 loss of function requires µ- and δ-opioid receptors**

 This paper continues the work of John Wood’s group in dissecting the role the mammalian voltage gated sodium channel *SCN9A*/Nav1.7 plays in pain. The human knock-out phenotype is pain free and anosmic. The mouse knock-out fails to thrive, assumedly because of a lack of weaning, but knock-out of the peripheral nociceptors (in the dorsal root ganglia and trigeminal ganglia) and sympathetic trunk recalculates the human pain free state. This naturally led pharmaceutical firms to seek Nav1.7 antagonists as a new class of analgesic - which should have worked for almost all types of pain and have the minimal side effect of temporarily losing the sense of smell. Many antagonists have been produced, but surprisingly they have little effect - why this should be the purpose of this paper.

 Previously the Wood team had shown that Nav1.7 knock-out mice had constitutional upregulation of the *Penk *gene (presumably this is also the case in humans, but this has yet to be shown, however they previously reported a single person with Congenital Insensitivity to Pain who did start to feel pain when given naloxone suggesting this is the case). Processing of the *Penk* gene produces a number of peptides including Met-enkephalin and Leu-enkephalin. They now explore if this is the cause of the painlessness seen in Nav1.7 knock-outs.

 The study reports complimentary mouse pain behavioural studies and cell biology of nociceptors derived from dorsal root ganglia. Firstly, mice with knock-out of either mu, delta or kappa opioid receptors were mated with those null for *SCN9A*/Nav1.7. The behavioural results showed that loss of mu and delta receptors together led to a substantial return of pain, but that kappa receptor had no role.  Secondly, naloxone (which blocks the effects of all opiates) reduced the analgesic effect seen in Nav1.7 null mice substantially but not completely. This data strongly suggests that a significant part of the analgesia seen in Nav1.7 knock-outs is mediated through increased opioid signalling through mu and delta opioid receptors.  

 Cell studies were performed by use of peripheral nociceptors derived from the mice used in the behavioural studies.  Thirdly, using various chemicals to manipulate intracellular sodium levels and the activities of Tetradotoxin sensitive voltage gated sodium channels (including Nav1.7) they found that Nav1.7 alone was responsible for raised *Penk* mRNA levels. And fourthly, that both *Penk* levels and that of the transcription factor *Nfat5 *were reduced by the increasing intracellular sodium concentrations and elevated by decreasing intracellular sodium concentrations. This data strongly suggested that intracellular sodium levels were responsible for the *Penk* upregulation when Nav1.7 is absent or non-functional. And fifthly, as *Nfat5* potential binds to the *Penk* 5’UTR they determined the effects of *NFAT5* knockout – in NFAT5 knock-out mice *PENK* levels were raised to a similar degree as seen in Nav1.7 knock-out mice, but surprisingly the NFAT5 knock-out mice had normal pain sensing. From this they concluded that increased *Penk* levels alone does not cause analgesia.  

 This paper presents data that Nav1.7 knock-out has complex consequences. Firstly, to achieve an analgesic effect complete knockout of Nav1.7 is needed – and maybe this explains why the anti-Nav1.7 analgesics used to date have had little effect. Secondly, the effects of Nav1.7 knock-out are significantly mediated through mu and delta opioid receptors. Thirdly, despite *Penk* being upregulated in Nav1,7 knock-down and being translated to produce endogenous endorphins, *Penk* upregulation alone does not cause analgesia. The authors hypothesise that it is local juxta-membrane sodium concentration changes controlled by Nav1.7 that effect mu and delta opioid function (and not the presence of Nav1.7 protein alone – as not all human SCN9A mutations effect Nav1.7 protein production and membrane localisation). And that locally decreased sodium levels lead to increased opioid receptor activity, and hence analgesia. For this reason they suggest that maybe pharmacological knock-down of Nav1.7 will not be possible, and the possibility of CRISPR-mediated gene knockout (in peripheral dorsal root ganglia) should be considered a possible treatment for extreme, intractable pain conditions. 

 This paper strongly supports the contention that Nav1.7 analgesia is mediated through peripheral nociceptors, with no evidence for a central effect.

 My only comment is that references should support the Discussion statement “ Evidence that co-administration of opioids with Nav1.7 antagonists….”.

I have read this submission. I believe that I have an appropriate level of expertise to confirm that it is of an acceptable scientific standard.
